# Mediastinal liposarcoma: a case report and review of the literature

**DOI:** 10.1186/s13256-023-04121-7

**Published:** 2023-08-31

**Authors:** Jamal Ataya, Ali Alakbar Nahle, Hussein Hamdar, Amjad Sikaria, Younes Souleiman

**Affiliations:** 1 Faculty of Medicine, University of Aleppo, Aleppo, Syria; 2 Faculty of Medicine, Damascus University, Damascus, Syria; 3 Thoracic Surgery, Damascus, Syria; 4 Thoracic Surgery, Alassad University Hospital, Damascus, Syria

**Keywords:** Mediastinal liposarcoma, Case report, Syria, Literature review, Cardiothoracic surgery

## Abstract

**Background:**

Mediastinal Liposarcoma (ML) is an exceedingly rare neoplasm, accounting for less than 1% of all liposarcomas. Surgical resection is the most effective therapeutic modality, while adjuvant radiation therapy may be recommended for unresectable tumors.

**Case presentation:**

This case report presents a rare case of a 52-year-old Syrian male patient with well-differentiated mediastinal liposarcoma (ML) who presented with exertional dyspnea, cough, and weight loss. Imaging studies revealed a large tumor causing extrinsic compression on the right lung, as well as compression of the heart and great vessels. CT-guided biopsy confirmed a diagnosis of well-differentiated liposarcoma, and the patient underwent surgical resection. The patient had a rapid postoperative recovery and was discharged on the fourth day post-operation, displaying an excellent postoperative status. The patient was followed up for 3 months postoperatively with no recurrence.

**Conclusion:**

This report highlights the significance of incorporating ML into the differential diagnosis of mediastinal masses, particularly in individuals presenting with exertional dyspnea and weight loss. Furthermore, this report provides a comprehensive review of previously reported cases of ML in the medical literature.

## Background

Liposarcomas are malignant neoplasms that originate from undifferentiated mesenchymal cells [1]. These tumors often develop in the lower extremities and retroperitoneal region. In contrast, mediastinal liposarcoma (ML) is an exceptionally rare neoplasm that accounts for less than 1% of all liposarcoma cases [1]. ML is classified into four distinctive histological subtypes, which include well-differentiated, myxoid, pleomorphic, and dedifferentiated liposarcoma [2]. The well-differentiated subtype is the most common subtype of ML and has a comparatively better prognosis than the dedifferentiated type [2]. The development of ML can be insidious and asymptomatic until the tumor reaches a critical size that causes symptoms associated with its growth and infiltration [3]. The clinical presentation may comprise a variety of nonspecific symptoms, including dyspnea, cough, and chest tightness [4, 5]. The most effective therapeutic approach for ML is surgery, with full surgical resection as the main therapy objective. Complete surgical excision is linked to a better prognosis, especially in cases of well-differentiated liposarcoma, and greater surgical resection correlates with better outcomes [2]. Adjuvant radiation therapy is recommended for patients with unresectable malignancies to improve local tumor control and lower the risk of local recurrence. However, role of chemotherapy and its significance in the management of ML is still debatable [2]. In this report, we discuss the case of a male patient, 52 years old, who had well-differentiated ML and had dyspnea at the time of his presentation. The patient's massive 25 cm tumor was successfully removed with surgery, which is still the backbone of care for this rare neoplasm. Additionally, we conducted a comprehensive review of previously reported cases of ML in the medical literature, which highlights the need for further research to optimize the management of this rare disease.

## Case presentation

**Fig. 1:**
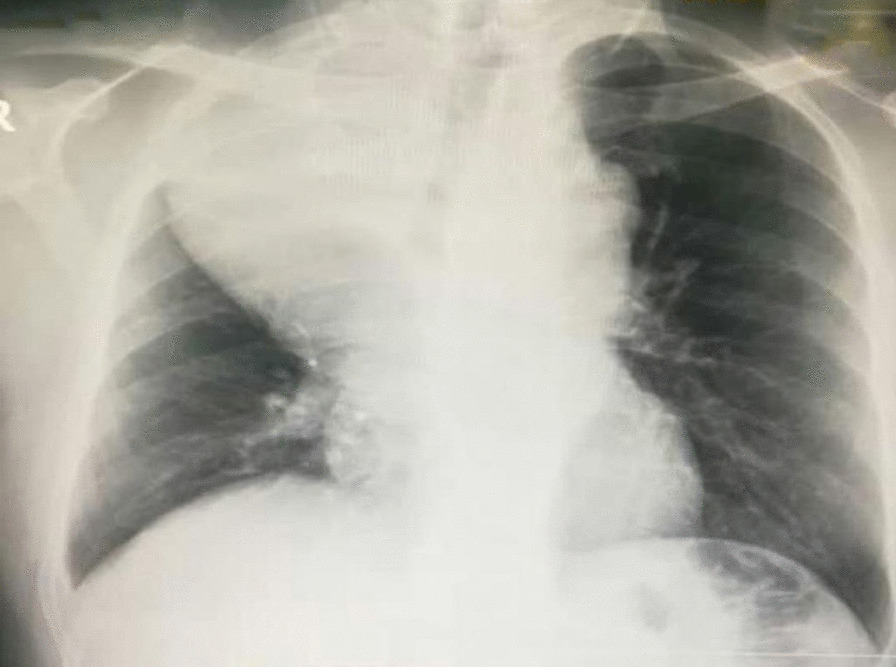
Presurgical Chest X-Ray showing a density occupying the right lung, suggestive of a possible mass

**Fig. 2:**
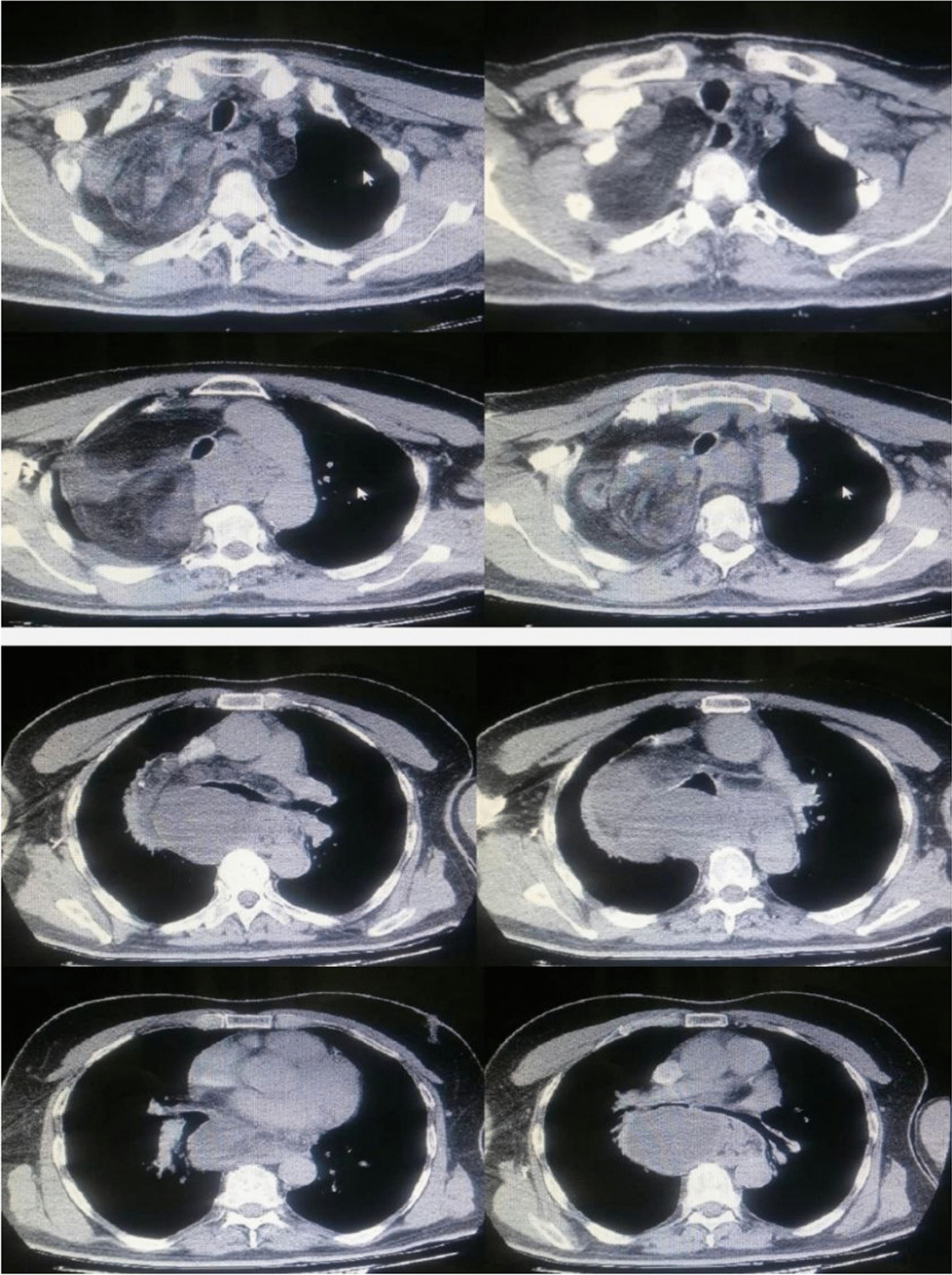
Presurgical Computed Tomography scan showing a mass with heterogeneous density involving the posterior mediastinum, spreading to the hilum, and upper lobe of the right lung

**Fig. 3:**
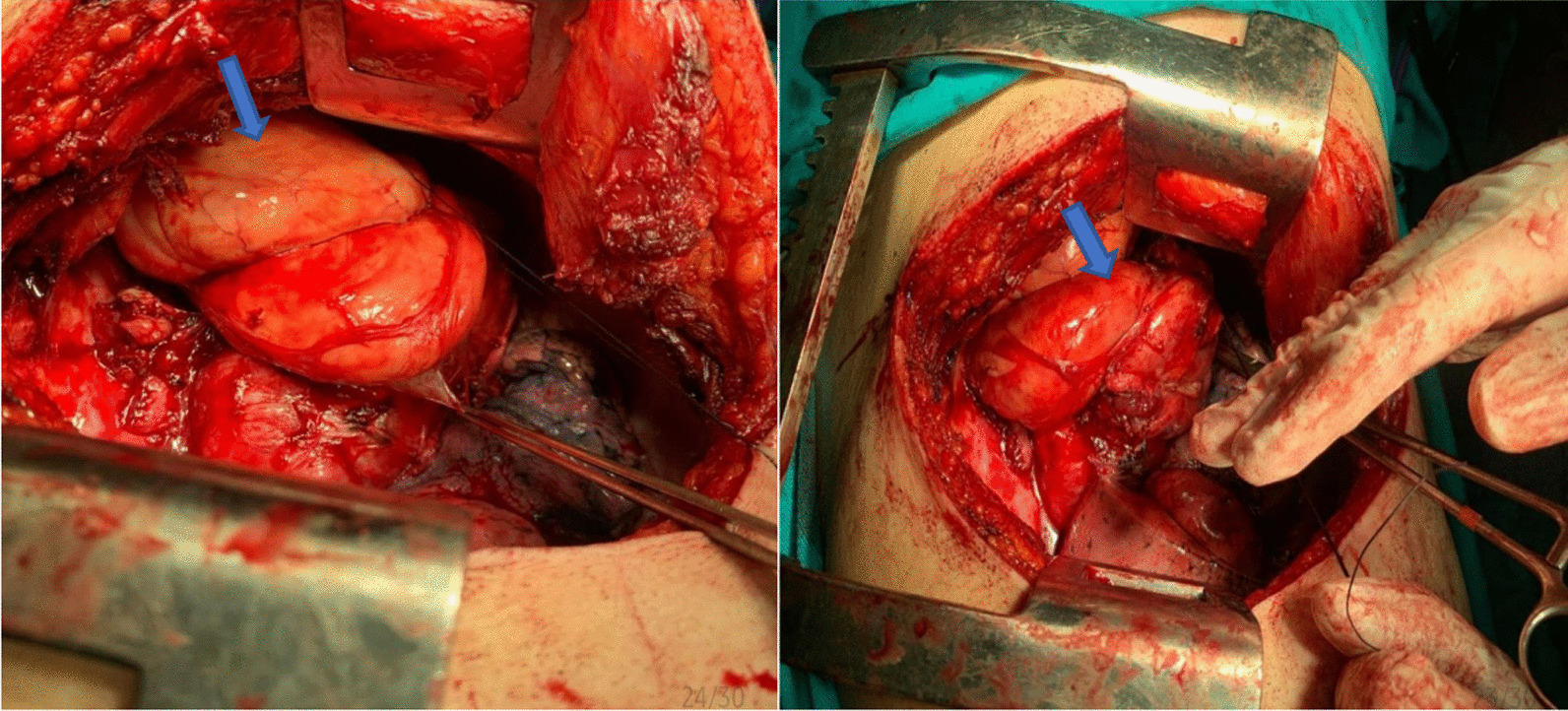
Lobulated mass during the surgery. The arrow indicates the location of the lobulated mass

**Fig. 4:**
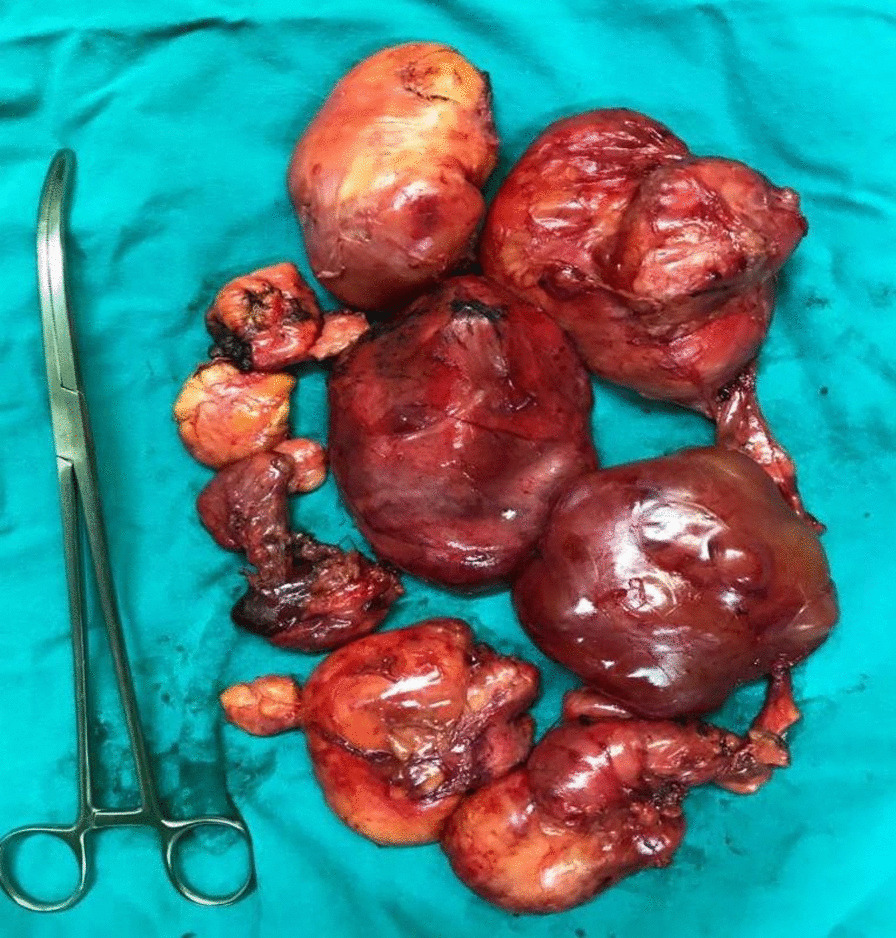
Specimen of the large posterior mediastinal tumor after resection

**Fig. 5:**
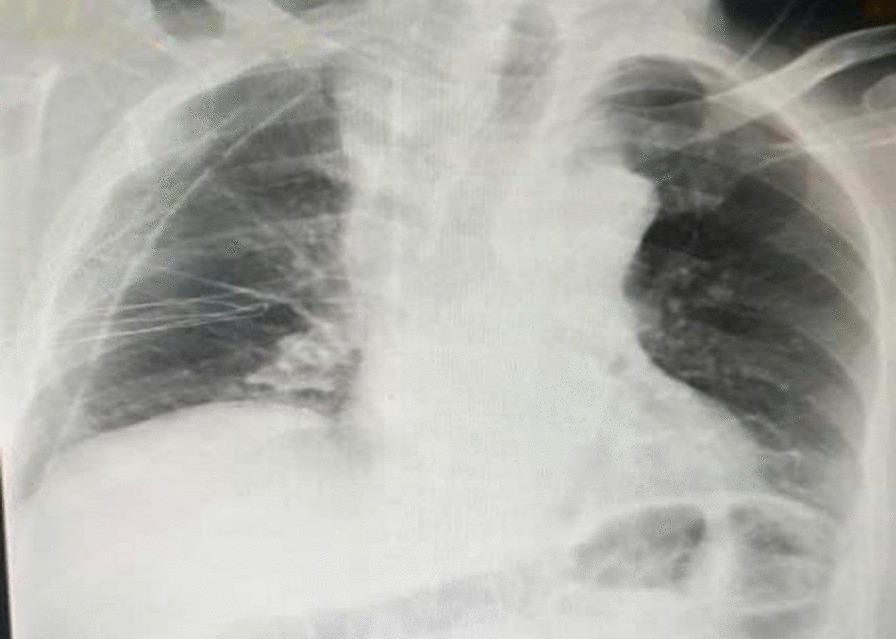
Follow up chest X-Ray demonstrating clear lungs and a central mediastinum of normal size

A 52-year-old Syrian male patient presented to the hospital with a 3-month history of progressive exertional dyspnea, accompanied by a dry cough, lethargy, and unintentional weight loss. No additional abnormal symptoms or medical history, such as hemoptysis, chest pain, or a history of infection, were noted. On physical examination, it was noticed that the upper lobe of the right lung had diminished breath sounds and was dull to percussion. Laboratory investigations such as cbc, crp, LFTs, spirometry, arterial blood gases and saturation tests were within normal range. Upon examination, a chest X-ray revealed the presence of a density occupying the right lung, suggestive of a possible mass (Fig. 1). However, a contrast-enhanced chest computed tomography (CT) scan demonstrated the existence of a significant mass with heterogeneous density that was located in the posterior mediastinum and extended to the hilum and upper lobe of the right lung (Fig. 2). The utilization of PET scans, EBUS, and MRI angiography was precluded in this case due to the substantial financial burden they imposed on the patient, exceeding their limited financial means. Furthermore, the restricted availability of these sophisticated diagnostic modalities further contributed to their omission. As an alternative, the patient underwent assessment utilizing a CT scan, which served as a substitute method for evaluation and diagnosis. This lesion was found to be the cause of extrinsic compression on the right lung. In addition, it was observed that the heart and great vessels, such as the aorta and superior vena cava, were subjected to compression displaying indistinct vascular borders, while the esophagus was anteriorly displaced. Bronchoscopy revealed severe extrinsic compression effects on the right main bronchus. The specimen comprises a mass measuring 25 × 10×8cm, displaying a lobulated and yellowish-white appearance similar to adipose tissue in texture. Microscopic examination revealed a well-differentiated liposarcoma with a low-grade malignant nature. The tumor exhibited localized areas of chondroid metaplasia and limited ossification. Abnormal cells were observed, featuring hyperchromatic nuclei, occasional prominent nucleoli, and increased mitotic activity. Focal regions demonstrated chondroid metaplasia and minimal ossification. Immunohistochemical analysis indicated positive expression of MDM2 and CDK4 in the tumor cells, while other markers, including S100 and desmin, were negative. The final pathological diagnosis is a well-differentiated, low-grade liposarcoma. In terms of TNM staging, the tumor was categorized as T2N0M0, indicating the absence of regional lymph node involvement (N0) and distant metastasis (M0). However, fibroadipose soft tissue with focal fibrosis were seen after a CT-guided biopsy, which is most consistent with well-differentiated liposarcoma. Consequently, surgical intervention was recommended. Under general anesthesia, the patient had a right thoracotomy with a fifth intercostal incision for the radical resection of the mediastinal lesion (Fig. 3a,b). The trachea and esophagus were examined and preserved during surgery, while the bulging azygos vein was ligated and removed. The lobular mass was resected, and it was composed of multiple nodules measuring up to 25 × 10x8cm, weighing 2250 g (Fig. 4). The final pathologic diagnosis confirmed well-differentiated low-grade liposarcoma. Patient’s postoperative recovery was rapid, and the procedure was well-tolerated. The chest tube was removed on the fourth day post-operation, with an output of 150 ml/24 h. After surgery, a chest X-ray revealed a clear lungs and a central mediastinum of normal size (Fig. 5). The patient was discharged on the fourth postoperative day and subsequently followed up 3 months later, exhibiting an excellent postoperative status.

## Discussion

Liposarcoma normally originates form mesenchymal cells [5]. ML is an extremely rare tumor representing 9% of all primary mediastinal sarcomas [6]. Due to rarity of this tumor, a PubMed search was conducted to perform a literature review over the last decade, using the keywords "Primary Mediastinal Liposarcoma" and "case report". The search yielded a total of 29 patients described in 18 articles within this timeframe [1–3, 5–19]. Table 1 was updated to include information about these 29 patients as well as our own case. Mediastinal liposarcoma according to the World Health Organization, is histologically classified into four subtypes: well-differentiated, myxoid, pleomorphic, and dedifferentiated liposarcoma [1]. The most common subtype is the well-differentiated type, which has a favorable prognosis, while the dedifferentiated subtype is associated with a poor prognosis [2]. In this study, the patient was diagnosed with the well-differentiated subtype, and our literature review showed that out of 13 patients with the same subtype [1, 6–8, 10, 16, 17, 19], ten had no recurrence after surgical intervention [6–8, 10, 17, 19]. This finding indicates a positive prognosis for this subtype.

**Table 1:** Review of research on the frequency, distribution, and therapy of mediastinal liposarcoma

Authors	Reported year	Age (years)/sex	Location of mass	Symptom	Size(cm)	Histologic analysis	Treatment	Follow up
Hirano	2014	53/F	Anterior	Chest discomfort	31 × 18 × 12	Well-differentiated liposarcoma	Surgical resection	No rec after 7 months
Fukuhara	2014	77/M	Anterior	Dyspnea of exertion	36 × 20 × 6.5	Well-differentiated liposarcoma	Surgical resection	No recurrence on 2 years
Luna-Martínez	2014	53/F	Anterior	Chest pain, cough and dyspnea of exertion	9 × 9	Pleomorphic liposarcoma	Surgical resection and radiotherapy (adjuvant)	Rec. after 16 years
Weissferdt	2014	52/F	Anterior	Dyspnea	35	Well differentiated liposarcoma	Surgical resection	Died due to the recurrence after 60 months
		68/F	Anterior	Dyspnea	13	Well differentiated liposarcoma		No rec. on 16 months
		57/M	Anterior	Dyspnea	10	Thymolipoma		No rec. on 36 months
Arrarás-Martínez	2015	68/M	Posterior	Dyspnea, dysphagia, palpitation	17.5 × 13x14	Mixed liposarcoma	Surgical resectionand chemotherapy(adjuvant)	Rec. after 1 year and died 3 months later
Mani	2015	28/F	Anterior	Asymptomatic	28.0 × 19.0 × 8.0	Well-differentiated liposarcoma	Surgical resection	No rec. (dates N/A)
Khan	2016	43/M	Anterior	Dyspnea and chest pain	4.8 × 12.6	Myxoid liposarcoma	Surgical resection and radiotherapy (adjuvant) and chemotherapy(adjuvant)	No recurrence on 10 months of follow up
Hamanaka	2016	74/M	Posterior	Dry cough	11 × 8 × 7	Dedifferentiated liposarcoma	Surgical resection	No rec. on 8 months
Zhao	2016	63/M	Posterior	Chest pain and dyspnea	24 × 22 × 16	Well-defferentiated liposarcoma	Surgical resection	1^st^:after 12 months:surgery
								2^nd^:after 2 yrs-died after 3 months
Muthukumar	2016	45/M	Posterior	Cough and dyspnea	14.5 × 13.5x6.5	Dedifferentiated myxoid liposarcoma	Surgical resection-radiotherapy (Adjuvent)	N/A
Mansuet-Lupo	2017	63/M	Anterior	Asymptomatic	N/A	Dedifferentiated liposarcoma	Surgical resection	N/A
Huang	2017	57 /M	Anterior	Dyspnea	27 × 20 × 15	Well-differentiated liposarcoma	Surgical resection	N/A
Weaver	2017	73/M	Posterior	Asymptomatic	12 × 10 × 6 30.5 × 16.5 × 8.5	Dedifferentiated liposarcoma	Surgical resection and chemo (neoadjuvant)	No rec. after 4 months
Miura	2018	45/F	Middle	Asymptomatic	12.7	Dedifferentiated liposarcoma	Surgical resection	After 12yrs:surgery
								No rec After 12 months
		62/F	Middle	Asymptomatic	12	Dedifferentiated liposarcoma	Surgical resection	After 28 months:radiation
								No rec. After 17 months
		81/M	Anterior	Asymptomatic	6.6 × 3	Dedifferentiated liposarcoma	Surgical resection	After 28 months:Surgery
								After 8 months:Radiation
		75/M	Posterior	Asymptomatic	20	Dedifferentiated liposarcoma	Surgical resection	No rec. After 3 months
		78/M	Middle and posterior	Dyspnea and hoarseness	11	Dedifferentiated liposarcoma	Radiation	Died two weeks later
Yang	2018	63/M	Anterior	Dyspnea and cough	22 × 16.0 × 11	Mixed: well and dedifferentiated liposarcoma	Surgical resection	No rec. after 2 yrs
Furlan	2020	33/F	Anterior	Chronic cough	18.5 × 15.0 × 6.0	Well differentiated liposarcoma	Surgical resection	No rec. on 1 year
Liu	2021	34/F	Anterior	Chest pain and shortness of breath	4 operations due to repeated recurrence: 1- N/A 2- 1.5 × 3 × 4 3- 10 × 9 × 3 4- 13 × 7 × 4	1-N/A 2- Partly myxoid and partly dedifferentiated liposarcoma 3- Myxoid liposarcoma 4- Well differentiated liposarcoma	1- Neoadjuvant radiotherapy and surgical resection 2- Surgical resection 3- Surgical resection 4- Surgical resection	1-First rec. after 10 years, 2- Second rec. after 20 years, 3-Third rec. after 3 months, No rec. on 2 years
Wong (6cases)	2022	76/F	Anterior	Dyspnea	10.5	Well-differentiated liposarcoma	Neoadjuvant radiotherapy and surgical resection	No recurrence on 2 years of follow up
		48/M	Anterior and Posterior	Chest congestion	40	Well-differentiated liposarcoma	Surgical resection	No recurrence on 4 years of follow up
		57/M	Anterior	Asymptomatic	10	Well-differentiated or dedifferentiated liposarcoma with myxoid features	Neoadjuvant radiotherapy and Surgical resection	No recurrence on 2 years of follow up
		68/M	Anterior	Dyspnea	29	Well-differentiated or dedifferentiated liposarcoma with myxoid features	Surgical resection and adjuvant radiotherapy	No recurrence on 1 year of follow up
		68/F	Anterior	Asymptomatic	9	Well-differentiated liposarcoma	Surgical resection	No recurrence on 1 year of follow up
		44/F	Posterior	Dysphagia	2.5	Dedifferentiated liposarcoma	Surgical resection and adjuvant radiotherapy	No recurrence on 7 years of follow up
Current case	2023	52/M	Posterior	Dyspnea and cough	25 × 10×8	Well-differentiated liposarcoma	Surgical resection	No recurrence on 3 months of follow up

The average age at which this tumor appears is 43 years old and it is uncommon among children and young adults [6] However, based on our analysis of 29 patients, along with our own case study, the average age of onset was found to be 58.7 years, which is significantly higher than previously reported findings. A clinical examination of 19 adult patients with primary mediastinal liposarcoma revealed that the incidence of this tumor did not appear to exhibit a marked preference for either gender, with 10 males and 9 females affected [20]. In our own case and literature review, out of 30 patients, 18 were males and 12 were females, indicating a slightly higher incidence among males. However, it should be noted that our review was limited to the last decade and therefore, may not be indicative of long-term study. According to Caraglia *et al.*, liposarcoma in the mediastinum is most frequently found in the posterior region and rarely found in the anterior region [6]. In contrast, our review of the literature showed that 19 out of 29 patients were diagnosed with anterior mediastinal liposarcoma [5–10, 12, 15–19]. Nevertheless, again our review is limited only for the last decade, and further research with long- term frame is necessary to draw more definitive conclusions.

In our analysis of previous cases, various symptoms were identified, including difficulty in breathing, coughing, trouble swallowing, chest discomfort, hoarseness, chest pain, and palpitations. The most common symptom was dyspnea, which was observed in 15 cases [1, 5, 8–12, 14, 16, 18, 19]. These symptoms are generally non-specific and occur due to the tumor in the mediastinum exerting pressure on or invading surrounding structures [4]. The patient we examined had dyspnea and coughing, which were caused by the compression of the tumor on the right lung. Typically, ML is detected incidentally during chest imaging for other reasons, with nonspecific findings [19]. Further diagnostic evaluation via CT is then performed to obtain detailed morphological information about the mass. Ultimately, a core needle or surgical biopsy is performed to establish a definitive diagnosis [19]. In our literature, 11 patients were found to have tumors of a giant size, equal to or greater than 20 cm [1, 3, 5–8, 10, 16, 19], while 13 had tumors of a relatively large size, equal to or greater than 10 cm [2, 10–14, 17–19], and 4 had small tumors, less than 10 cm in size[2, 9, 19]. Our case, on the other hand, featured a giant tumor that measured 25 cm. This review indicates that three patients with giant tumors as well as three with large tumors did not display any symptoms [2, 3, 6, 19]. This highly supports a previous finding that tumors can attain a substantial size before presenting any symptoms [3].

Open surgical resection with negative margins is the established approach [2]. Based on the literature, surgical resection is the primary intervention for all cases, underscoring its significance in treatment. Furthermore, Lui *et al.* reported a case of a patient who suffered from recurrence thrice and underwent surgical management each time, which underscores the value of surgery even with multiple resections [18]. The utilization of adjuvant radiation therapy can boost local tumor control and decrease the likelihood of local recurrence, especially in patients with an unrespectable tumor. Nevertheless, the utility of chemotherapy in managing ML remains a contentious topic [2]. Among the cases identified in the literature, eight patients underwent radiotherapy as an adjuvant therapy, with follow-up periods ranging from 10 months to 16 years [9, 12, 14, 18, 19]. Five patients had no recurrence during follow-up periods ranging from 10 months to 7 years [12, 19]. However, late follow-up exceeding 10 years revealed recurrence in two patients, as reported by Lui *et al.* and Luna-Martínez *et al.*, respectively [9, 18]. This indicates that adjuvant radiotherapy is essential in minimizing the risk of recurrence, although late follow-up (more than 10 years) is necessary to detect any tumor recurrence. With respect to chemotherapy, three patients underwent adjuvant and neoadjuvant therapy [3, 11, 12]. Among them, two showed no recurrence after follow-up periods of only 4 and 10 months [3, 12], whereas the third developed another tumor after one year of follow-up and passed away 3 months later [11]. As a result, chemotherapy is less beneficial than radiotherapy in managing ML. In our patient, we monitored him for three months only after surgery but lost follow-up afterwards. In managing this case, we encountered significant difficulties stemming from the lack of resources. One major challenge we faced was the limited availability and high cost of advanced imaging modalities such as PET scans, EBUS, and MRI angiography, which were crucial for accurate diagnosis and staging. To overcome this limitation, we relied on alternative diagnostic methods, primarily CT scans, which provided essential information within the available means. Additionally, we employed meticulous clinical judgment and interdisciplinary collaboration to compensate for the absence of certain tests, ensuring comprehensive evaluation and optimal patient care despite resource constraints. These adaptive strategies exemplify the resilience and resourcefulness required in navigating challenging healthcare settings with limited resources.

## Conclusion

In conclusion, primary mediastinal liposarcoma is a rare mesenchymal tumor originating from adipose tissue in the mediastinum. The dedifferentiated subtype of this tumor is linked to poor prognoses, whereas the well-differentiated subtype is associated with more favorable prognoses. The preferred method of managing all cases is surgical resection with negative margins, with adjuvant radiation therapy being essential to reduce recurrence risk. The effectiveness of chemotherapy in ML treatment is controversial and has limited benefits. The average age of ML patients, gender prevalence, and tumor localization have yielded conflicting results, emphasizing the need for further research with larger sample sizes to yield more conclusive findings.

## Data Availability

Not Applicable.

## Abbreviation

ML: Mediastinal liposarcoma

## References

[1] Zhao C, Zhang F, Zhang X, Tu S, Wu Z, Li X, Xiang Y, Zheng C, Zeng Q (2016). Recurrent primary mediastinal liposarcoma: a case report. Oncol Lett.

[2] Miura K, Hamanaka K, Matsuoka S, Takeda T, Agatsuma H, Hyogotani A, Ito KI, Nishimaki F, Koizumi T, Uehara T (2018). Primary mediastinal dedifferentiated liposarcoma: five case reports and a review. Thorac Cancer.

[3] Weaver HL, Preston SD, Wong HH, Jani P, Coonar AS (2018). Surgical resection of a massive primary mediastinal liposarcoma with cervical extension. Ann R Coll Surg Engl.

[4] Barbetakis N, Samanidis G, Paliouras D, Boukovinas I, Kiziridou A, Tsilikas C (2008). A rare cause of mediastinal mass: Primary liposarcoma. J BUON.

[5] Yang YS, Bai CY, Li ZC, Li WJ, Li Y (2018). Giant primary liposarcoma of the anterior mediastinum: a case report. Medicine (Baltimore).

[6] Mani VR, Ofikwu G, Safavi A (2015). Surgical resection of a giant primary liposarcoma of the anterior mediastinum. J Surg Case Rep.

[7] Hirano Y, Yamamoto H, Ichimura K, Toyooka S, Miyoshi S (2014). Surgical resection of a massive primary mediastinal liposarcoma using clamshell incision combined with lower median sternotomy: report of a case. Ann Thorac Cardiovasc Surg.

[8] Fukuhara S, Dimitrova KR, Geller CM, Hoffman DM, Ko W, Tranbaugh RF (2014). Progressive dyspnea in patient with large mediastinal mass. J Cardiothorac Surg.

[9] Luna-Martínez J, Molina-Ramírez D, Mata-Quintero CJ, García-Arrona LR, Peña-Rosas DP, Mondragón-Pinzón EE. Liposarcoma mixoide de mediastino anterior. Reporte de caso y revisión de la bibliografía [Myxoid liposarcoma of the anterior mediastinum. A case report and bibliography review]. Cir Cir. 2014;82(2):177–182. [**Spanish**] 25312317

[10] Weissferdt A, Moran CA (2014). Lipomatous tumors of the anterior mediastinum with muscle differentiation: a clinicopathological and immunohistochemical study of three cases. Virchows Arch.

[11] Arrarás-Martínez MJ, Rieger-Reyes C, Panadero-Paz C, Landa-Oviedo HS, García-Tirado J (2015). Giant primary mediastinal liposarcoma: a rare cause of atrial flutter. Asian Cardiovasc Thorac Ann.

[12] Khan MH, Kashif R, Rahim Khan HA, Fatimi SH (2016). Myxoid liposarcoma originating in the anterior mediastinum. J Ayub Med Coll Abbottabad.

[13] Hamanaka K, Ohashi M, Nakamura T (2016). Primary mediastinal dedifferentiated liposarcoma resected by lateral thoracotomy with video-assisted thoracoscopic surgery. J Surg Case Rep.

[14] Muthukumar S, Rajendiran S, Damodharan J (2017). Primary dedifferentiated massive mediastinal liposarcoma weighing 53 kg. Asian Cardiovasc Thorac Ann..

[15] Mansuet-Lupo A, Lococo F, Larousserie F, Alifano M, Saliceti R (2017). Dedifferentiated primary mediastinal liposarcoma mimicking a thymic tumor. Pathologica.

[16] Huang W, Jiang GN (2017). Resection of giant mediastinal liposarcoma via '⊣ shape' incision. J Surg Case Rep.

[17] Furlan K, Miller I, Rohra P, Mir F, Ocampo Gonzalez FA, Gattuso P (2020). Well-differentiated liposarcoma primary from thymic stroma: case report and literature review. Exp Mol Pathol..

[18] Liu Z, Du M, Liang Y, Gao Y (2021). Multiple surgical excision for recurrent primary mediastinal liposarcoma. Ann R Coll Surg Engl.

[19] Wong GS, Bass D, Chen IY, Thomas R, Velez MJ, Hobbs SK (2022). Imaging and clinical findings in a series of six cases of rare primary mediastinal liposarcoma. Radiol Cardiothorac Imaging..

[20] Su K, Cheng GY, Liu XY, Meng PJ, Zhao J, Chen XJ, He J (2012). Clinical analysis of 19 cases of adult primary mediastinal liposarcoma. Zhongguo Yi Xue Ke Xue Yuan Xue Bao.

